# Engineering soil organic matter quality: Biodiesel Co-Product (BCP) stimulates exudation of nitrogenous microbial biopolymers

**DOI:** 10.1016/j.geoderma.2015.06.006

**Published:** 2015-12

**Authors:** Marc A. Redmile-Gordon, Richard P. Evershed, Alison Kuhl, Elena Armenise, Rodger P. White, Penny R. Hirsch, Keith W.T. Goulding, Philip C. Brookes

**Affiliations:** a Dept. of Sustainable Soils and Grassland Systems, Rothamsted Research, Harpenden, Hertfordshire AL5 2JQ, UK; b Organic Geochemistry Unit, Bristol Biogeochemistry Research Centre, School of Chemistry, University of Bristol, BS8 1TS, UK; c School of Energy, Environment and Agrifood, Building 52a, Cranfield University, Cranfield, Bedfordshire MK43 0AL, UK

**Keywords:** Extracellular polymeric substances, EPS matrix, Exocellular amino acids, Exopolysaccharide, Exopeptide, Protein dynamics, Nitrate, Glycerol

## Abstract

Biodiesel Co-Product (BCP) is a complex organic material formed during the transesterification of lipids. We investigated the effect of BCP on the extracellular microbial matrix or ‘extracellular polymeric substance’ (EPS) in soil which is suspected to be a highly influential fraction of soil organic matter (SOM). It was hypothesised that more N would be transferred to EPS in soil given BCP compared to soil given glycerol. An arable soil was amended with BCP produced from either 1) waste vegetable oils or 2) pure oilseed rape oil, and compared with soil amended with 99% pure glycerol; all were provided with ^15^N labelled KNO_3_. We compared transfer of microbially assimilated ^15^N into the extracellular amino acid pool, and measured concomitant production of exopolysaccharide. Following incubation, the ^15^N enrichment of total hydrolysable amino acids (THAAs) indicated that intracellular anabolic products had incorporated the labelled N primarily as glutamine and glutamate. A greater proportion of the amino acids in EPS were found to contain ^15^N than those in the THAA pool, indicating that the increase in EPS was comprised of bioproducts synthesised de novo. Moreover, BCP had increased the EPS production efficiency of the soil microbial community (μg EPS per unit ATP) up to approximately double that of glycerol, and caused transfer of 21% more ^15^N from soil solution into EPS-amino acids. Given the suspected value of EPS in agricultural soils, the use of BCP to stimulate exudation is an interesting tool to consider in the theme of delivering sustainable intensification.

## Highlights

• BCP made from waste oils (BCP_R_) resulted in the highest EPS-production efficiencies.
• BCP_R_ resulted in the greatest ^15^N accumulation in *extracellular* bioproducts.
• Glycerol resulted in the greatest ^15^N accumulation in *intracellular* bioproducts.
• δ^15^N of extracellular amino acids shows that resin extracts EPS synthesised de novo.
• Leucine in EPS was the most enriched soil amino acid measured (up to 29% ^15^N).

## 1 Introduction

The greatest challenge for soil science over the next few decades is to help alleviate competing demands on soil for security of water, food, fuel, fibre, and ecosystem pressures without expanding the total area of soil under exploitation (Tilman et al., 2011). The need to engineer soils to deliver sustainable intensification objectives is therefore increasingly urgent. It is also increasingly achievable as the shortfalls between soil capability and actual condition become more apparent (McBratney et al., 2014). We propose that some alleviation to the pressure on soil functions may be found through employing native soil microbial populations to better utilise the waste streams from otherwise competing industries. If successful, this approach will challenge the simplistic perception that one agricultural output (e.g., food) necessarily conflicts with another (e.g., fuel). The global demand for biodiesel is driven by the need to reduce dependence on fossil fuels which exacerbate climate change. However, the energy embodied in fertiliser use for crop growth is significant, with fertiliser N production estimated to account for 2% of the total global energy budget (Smith et al., 2012). Moreover, the variable efficiencies of biodiesel production are strongly affected by subsequent loss of fertiliser N from soils, especially as N_2_O which has about 300 times the global warming potential of CO_2_ on a mole for mole basis (Crutzen et al., 2008). Nitrogen dynamics (affected by soil management) are therefore central to the debate surrounding the efficiency of biofuel production and life cycle analysis (LCA) of the biodiesel industry.

Biodiesel Co-Product (BCP) contains many biodiesel-processing residues, being a water soluble mixture of glycerol, salts of fatty acids, methyl esters, mono- and di-glycerides, potassium (or sodium) hydroxide, methanol and water. After production, BCP can be refined, for example to isolate glycerol for industrial/cosmetic uses. This was once an attractive option, but while it is possible to obtain glycerol (> 95% purity) from BCP — this requires financial outlay for refining facilities, is energy intensive, and so can be counter-productive (Raghareutai et al., 2010). More direct uses of BCP offer intriguing environmental prospects, e.g., as a component of soil treatments to engineer improved (or repair lost) soil functions. The application of BCP to soil as a substrate for the native soil microbial biomass was previously found to be > 99% effective in causing nitrate (NO_3_) capture from soil solution and thus preventing loss from surface horizon (23 cm) in winter (Redmile-Gordon et al., 2014a). BCP therefore has considerable potential for improving nitrogen use-efficiency and limiting the environmental damage caused by ‘leaky’ agriculture.

While the previous study demonstrated that BCP prevented depletion of soil N, only about half of the N prevented from leaching could be accounted for by the microbial biomass N. The net gain in soil N observed suggested that a large alternative (*extracellular*) repository of N was created. Besides reducing losses of N, the agricultural advantages of increasing the mass of the extracellular matrix are suspected to include: (i) improving soil aggregate stability (Watts et al., 2005; Redmile-Gordon et al., 2013), (ii) increasing survivability of the soil microbial biomass under drought conditions (Or et al., 2007a) and (iii) improving overall water retention in sandy or dry soils (e.g., Rosenzweig et al., 2012). The recent development of methods to extract and quantify changes in the mass of the extracellular microbial matrix, or ‘extracellular polymeric substances’ (EPS; Redmile-Gordon et al., 2014b) mean that more descriptive investigations of soil organic matter (SOM) are now possible, and therefore of value in the investigation of ‘organic engineering’ methods which aim to cause the redirection of soil microbial resources into the extracellular matrix.

An unpublished study of the soils treated by Redmile-Gordon et al. (2014a) indicated that BCP had indeed increased the production of EPS in soil. In the present study we hypothesise that, in a similar soil, increased allocation of the NO_3_-N pool to EPS-N would arise from BCP additions (in comparison to no amendment). Since the wide variety of labile carbon compounds in BCP is likely to support a diversity of microbial activity and niches, we also hypothesised that this soil amended with BCP would induce greater production of EPS-protein and EPS-amino acids (per unit of microbial biomass) than soil amended with a simple carbon source (glycerol). These hypotheses were investigated through ^15^N-stable isotope probing of extracellular amino acids produced de novo, which were performed alongside more rapid colorimetric techniques. Increased abundance of heavy isotopes in the EPS was taken as evidence that the applied NO_3_-N had been immobilised from the soil solution as opposed to N derived from the atmosphere or soil organic matter. The content of extracts of EPS were also compared to the total hydrolysable amino acid pool (THAA; Knowles et al., 2010) to provide a contrast with the amino acid profile of the more general soil organic matter (SOM) pool.

Since the purpose behind engineering an increased EPS production and reduced N loss in soil is towards delivering sustainable intensification, we also compared responses to BCP made from recycled vegetable oils (BCP_R_; from a variety of restaurants) with BCP produced from food-grade ‘virgin’ oilseed rape (BCP_V_). The aim of this additional comparison was to contribute towards future development of more complete LCA's of biodiesel made from oil-crops grown for that sole purpose (BCP_V_) and biodiesel made from recycled oils (BCP_R_).

## 2 Materials and methods

### 2.1 Preparation of Biodiesel Co-Product (BCP)

An alkali-catalysed transesterification of vegetable oils produced approximately 20 L of methanol rich BCP per 100 L biodiesel after separation. A catalytic quantity of 7 g pure KOH L^− 1^ oil (corrected quantity for actual purity of 82%) plus a titrated quantity (2.5 g of 82% KOH to neutralise free fatty acids in the feedstock oil) was used for transesterification. Excess methanol was evaporated from 1 L of BCP in a 5 L beaker placed on a hotplate for 2 h (stirred at 70–80 °C). The organic constituents of BCP were determined externally by Harvest Energy Ltd. (York House, London), and are provided in Table 1. Total C and N were determined by combustion analysis (LECO). Inorganic content was given by analysis of duplicates using ICP-MS after digestion of subsamples of BCP in a mixture of nitric and perchloric acid (Zarcinas et al., 1987). Of the 20 elements measured, BCP contained lower concentrations than material harvested from a non-contaminated grassland at Rothamsted (UK), with elements As, Cd, Cr, Ni and Pb always below 1 ppm (Supplementary Table 1). Importantly, the total N contents of both BCP's were negligible (< 0.0005%; below the limit of detection).

### 2.2 Soil sampling

Soil was sampled on 22^nd^ February 2012 from the Highfield Conversion Experiment at Rothamsted Research. This soil is a Batcombe Series (Soil Survey of England and Wales) fine silty loam, over clay drift with siliceous stones: roughly equivalent to a Chromic Luvisol as defined in the FAO soil classification system (Avery and Catt, 1995). Plot 12 (historically managed as grassland and switched to arable management in 2008) was sampled randomly with a 2.5 cm auger to a depth of 23 cm and sieved < 2 mm. Visible root material and organisms were removed. This specific soil management of grassland to arable was selected due to the intrinsic presence of a large pool of humified (and refractory) organic matter. The soil was pre-incubated at 25 °C in the dark for one week to allow the mineralisation of substrates brought into fresh contact with microorganisms during sieving. Sand silt and clay proportions were 11, 66 and 23%, respectively (Watts and Dexter, 1997). The pH of soil in water was 6.50. The soil contained little easily available C (by design) due to the pre-incubation period and sampling time (late February 2012). Inorganic N availability was also likely to be low after exposure to 5-months rainfall and N leaching following the wheat harvest in August 2011 (Redmile-Gordon et al., 2014a).

### 2.3 Treatments

Glass equipment was used throughout. Treatments (Table 2) were prepared in water (18 MΩ resistivity). K^15^NO_3 (aq)_ was added to glycerol (39.13% C; Molecular biology grade, VWR Prolabo product# 444482 V), BCP_R_ (51.52% C), or BCP_V_ (39.17% C). BCP_R_ was produced from reclaimed cooking oils and BCP_V_ produced from the oil of pressed oilseed-rape. Since K^+^ is a major component of BCP, and was always present in excess of biological requirement in treatments including BCP, we used it as the cation to balance counter-ion concentrations in amendments (OH^−^, Cl^−^). BCP is variably alkaline and therefore the BCP treatments were adjusted to pH 8 using HCl (1 M). Further KCl (1 M) was added to BCP_R_, control and glycerol treatments to balance the concentration of Cl^−^. The glycerol and water treatments were brought to pH 8 using 0.1 M KOH. The remaining water was then added. Soil moisture was brought to 60% of its water holding capacity (WHC) by addition of each amendment. Each portion of moist soil was placed into 125 mL glass beakers thus forming 12 microcosms. The four aqueous solutions (control containing only water and N) were mixed immediately before drip-application to soil. All microcosms were thus provided with 150 μg ^15^N g^− 1^ soil as 99.7 Atom % K^15^NO_3_ (Cambridge Isotope Laboratories, lot# I-15284 L, MA USA). With the exception of the controls, all treatments gave 3 mg Cg^− 1^ soil, (either as BCP_V_, BCP_R_, or glycerol) with a substrate C/N of 20. Each microcosm was then placed in a desiccator (each desiccator forming 1 block of the treatment design) and experimental units randomly distributed within. A vial of soda lime was placed in the centre of each desiccator to prevent CO_2_ accumulation. Silica gel in the base of each desiccator (380 g dry weight) was changed every 48 h in order to progressively dry the soil, which favours the production of EPS (drought was previously seen to maximise EPS production in sand; Roberson and Firestone, 1992). Soil was allowed to dry to 20% WHC before being re-moistened to 40% WHC with deionised water: this was required twice over the experimental period of 10 days. Besides previously being observed to trigger an EPS response, moisture fluctuations ensured more complete circulation of solutes as would occur naturally in an exposed system.

### 2.4 EPS extraction and colorimetric analyses

EPS was extracted as described by Redmile-Gordon et al. (2014b). Briefly, after the removal of soluble microbial products from the soil with dilute CaCl_2_ (0.01 M), EPS was extracted using cation exchange resin (CER) following separation by centrifugation. Three technical replicates of moist soil (2.5 g dry weight equivalent) were used to estimate microbial ATP before extraction and three were subjected to extraction of EPS. ATP concentrations of the extracted soils were compared with those of the non-extracted to estimate the extent of cell-lysis. ATP of the microbial biomass was quantified using the method described by Redmile-Gordon et al. (2011). EPS-polysaccharide content was estimated colorimetrically using the method of DuBois et al. (1956) and EPS-protein using a Lowry microplate assay modified for use with soil extracts (Redmile-Gordon et al., 2013).

### 2.5 Polypeptide hydrolysis and purification

20 mL quantities were taken from each technical replicate of EPS extract, then combined and freeze-dried to provide lyophilate of 60 mL extract per biological replicate (or microcosm). Lyophilate was transferred to 10 mL thick-walled pyrex digestion tubes (threaded apertures with PTFE sealed caps) using 3 successive 1 mL aliquots of 0.1 M HCl. The HCl was then removed under a stream of N_2_ at 60 °C. Norleucine (20 μg) was added to each EPS extract and each soil (soils previously air-dried and milled) to serve as an internal standard (Jim et al., 2003). Amino acids in the soil (THAA) and EPS-AA were liberated by hydrolysis with 5 mL of 6 M HCl at 100 °C for 18 h. Asparagine (Asn) and glutamine (Gln) could not be distinguished from their corresponding acids (Aspartate and Glutamate) as the amides were destroyed during hydrolysis (Roberts and Jones, 2008). The sums of original amides and deamidated amino acids are thus referred to as Glx, and Asx, respectively. All glassware had been washed with detergent (Decon 90; Decon Laboratories Ltd.), rinsed with double distilled water (DDW) then baked in a furnace at 450 °C for ≥ 8 h. Double distilled water (DDW) was produced using a Bibby Aquatron still. Water was also extracted with dichloromethane (DCM) before use.

Impurities were removed from the hydrolysate by washing through cation exchange resin (Dowex 50X-W8; Sigma Aldrich, Dorset, UK). Resin was pre-soaked for 12 h in 3 M NaOH to remove contaminants. NaOH was then removed and resin washed in DDW (× 6). To ensure all cation exchange sites were saturated with H^+^, resin was steeped in 6 M HCl for ≥ 24 h. Aliquots of resin (1 mL) were transferred to flash columns, and washed (6 × 2 mL DDW). Each soil hydrolysate was added to a dedicated column for the exchange of AAs. Non-affixed solutes were eluted to waste with 4 × 2 mL DDW before displacing the AAs with 6 × 1 mL 2 M NH_4_OH. The resulting solution was blown down to dryness under N_2_ at 60 °C.

### 2.6 Derivatisation and GC–FID analyses

A solution of AA standard was prepared containing 1 mg of each AA mL^− 1^ 0.1 M HCl (isotope ratios at natural abundance) firstly to validate retention times and secondly to confirm fragmentation weights and ratios given by mass-spectrometry (Section 2.7). All solvents were HPLC grade. N-acetyl, O-isopropyl derivatives of hydrolysed AAs were prepared firstly by conversion to their respective isopropyl esters followed by N-acetylation as described by Corr et al. (2007). Preparations were cleaned by phase extraction with saturated aqueous NaCl and remaining solvent was evaporated under a gentle stream of N_2_ at 20 °C. Potential residual water was removed by sequential blow down in DCM. Derivatised samples were capped, sealed with PTFE and stored in a freezer at − 20 °C for subsequent analysis.

In preparation for GC–FID, samples were suspended in approximately 100 μL ethyl acetate and analysed using a Hewlett-Packard 5890 gas chromatograph fitted with a flame ionisation detector (FID) and VF-23MS capillary column (3-cyanopropylpolysiloxane stationary phase, 60 m × 0.32 mm internal diameter; 0.15 μm film thickness; Varian Ltd., Oxford, UK). About 0.5 μL of each sample was introduced using direct on-column injection. The carrier gas was H_2_ with a flow rate of 3 mL min^− 1^; head pressure 10 PSI. Initial oven temperature was 40 °C and held for 1 min before heating at 15 °C min^− 1^ to 120 °C (no hold), then 3 °C min^− 1^ to 190 °C (no hold) and finally 5 °C min^− 1^ to 250 °C and held for 20 min. The concentration of each AA was calculated by comparison of peak area with the internal standard (norleucine) and adjusted for flame response of each AA as given from the mixed standard.

### 2.7 GC–combustion–isotope ratio MS (GC–C–IRMS) analyses and GC–MS

Nitrogen stable isotope compositions were determined by GC–C–IRMS using a Thermoquest Trace GC connected to a DeltaPlus XP via a GC combustion III interface. A similar VF-23MS capillary column was used as for GC–FID (see above). Samples were introduced to a PTV ‘splitless’ injector via auto-sampler. δ^15^N values needed no adjustment for addition of the derivative (NAIP) as it contains no nitrogen (Corr et al., 2007). Compound identities were verified using a Trace GC/MS (Thermo Finnigan Ltd., Hemel Hempstead, UK) also equipped with a VF23-MS column, carrier gas was helium (2 mL min^− 1^). Samples were introduced in splitless mode (inlet temp 250 °C). Source held at 200 °C, ionisation energy 70 eV with mass analyser scanning the range *m*/*z* 50–650. The GC oven was programmed as for GC–FID (described in Section 2.6). Mass spectra were consistent with those normally observed for NAIP-derivatives of the amino acids described (or isomers), i.e., the mass of the ionised AA NAIP and fragmentation resulting from the loss of propyl [CH(CH_3_)_2_]/*O*-propyl [OCH(CH_3_)_2_]/COO-propyl [CO_2_CH(CH_3_)_2_] and/or one of acetyl [COCH_3_]/[CH_3_] and one or two H^+^. Elution order was confirmed as following that described by Corr et al. (2007).

### 2.8 Statistical analyses

Treatments were analysed for their contributions to variables of AA concentration and nitrogen isotope compositions. For direct comparison of nitrogen isotope compositions between the EPS and THAA extracts ANOVA was performed using Genstat software to analyse the experimental structure (blocks/treatment/extraction-method/AA). This enabled statistical distinction between the two pools of AAs to identify which pool contained proportionally greater ^15^N (from KNO_3_): THAA vs EPS-AA. Subsequently, THAA and EPS were analysed separately as different pools to quantify the likelihood of treatment effects and AA interactions (the ‘interaction’ being used to indicate AA specific changes in the AA profile or ^15^N enrichments in response to treatment). Since residual variability increased with concentration, the data were transformed (log_10_) to identify statistical significance of effects. ^15^N % enrichment is presented on the natural scale as no systematic increase in residual variability was observed. All analyses were performed using Genstat software (15th edition).

## 3 Results

### 3.1 GC–FID quantitative analyses

**Fig. 1:**
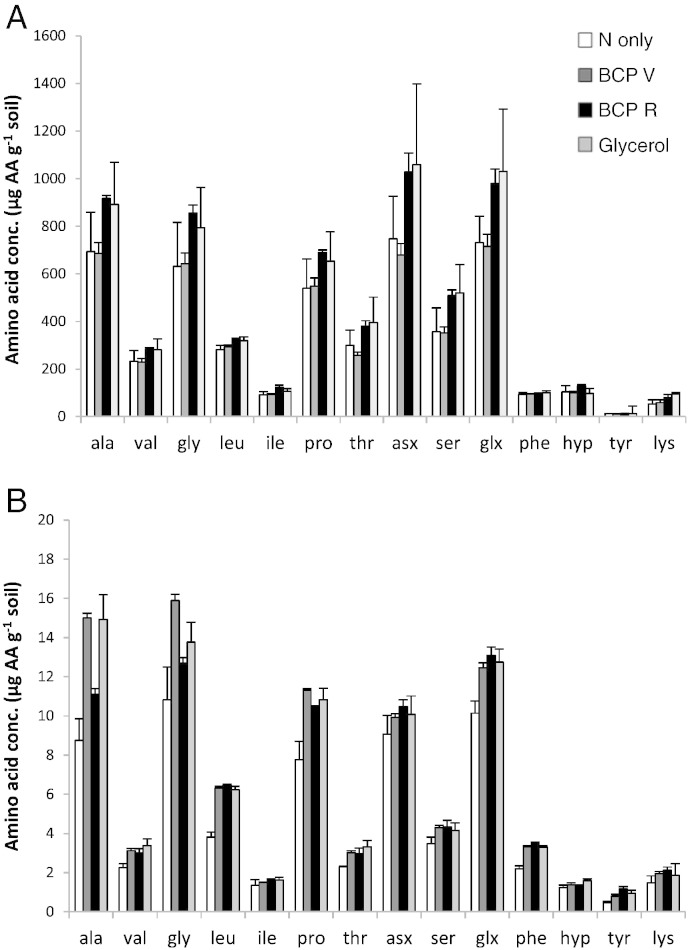
A: Total hydrolysable amino acids (THAAs), GC–FID quantified; data presented on the natural scale ± std. error; ANOVA of transformed data indicated no statistically significant treatment effect (p = 0.363). B: EPS amino acids, GC–FID quantified; data presented on the natural scale ± std. error; ANOVA of transformed data indicates treatment effects (Table 3; p = 0.024).

While glycerol and BCP_R_ both appeared to potentially increase the concentrations of amino acids (AAs) in the total hydrolysable pool (THAA; Fig. 1A) ANOVA revealed no statistically significant effect of treatment (P = 0.363). The only statistically significant effect upon concentration was the identity of the AA being quantified (p < 0.001). In contrast, quantitative analysis of the EPS showed a statistically significant effect of treatment (Fig. 1B; p = 0.02). An interaction effect was identified between treatments and AAs which was statistically significant (p = 0.001; Supplementary Table 3). This means that treatments exerted bias, augmenting some residues more than others (creating contrasting ‘AA profiles’). Comparing EPS-AA concentrations per unit *soil* determined by GC–FID alone, shows no statistically significant differences in mean EPS-AA concentrations between treatments BCP_V_, BCP_R_, and glycerol (Supplementary Table 3) — with all giving statistically significant increases in EPS-AA relative to ‘N only’ (l.s.d. = 0.095 μg g^− 1^ soil; data transformed log_10_). However, Section 3.3 shows contrasting results when investigating EPS per unit microbial biomass ATP, i.e., as ‘EPS production efficiency’.

### 3.2 Treatment effects on nitrogen stable isotope compositions

**Fig. 2:**
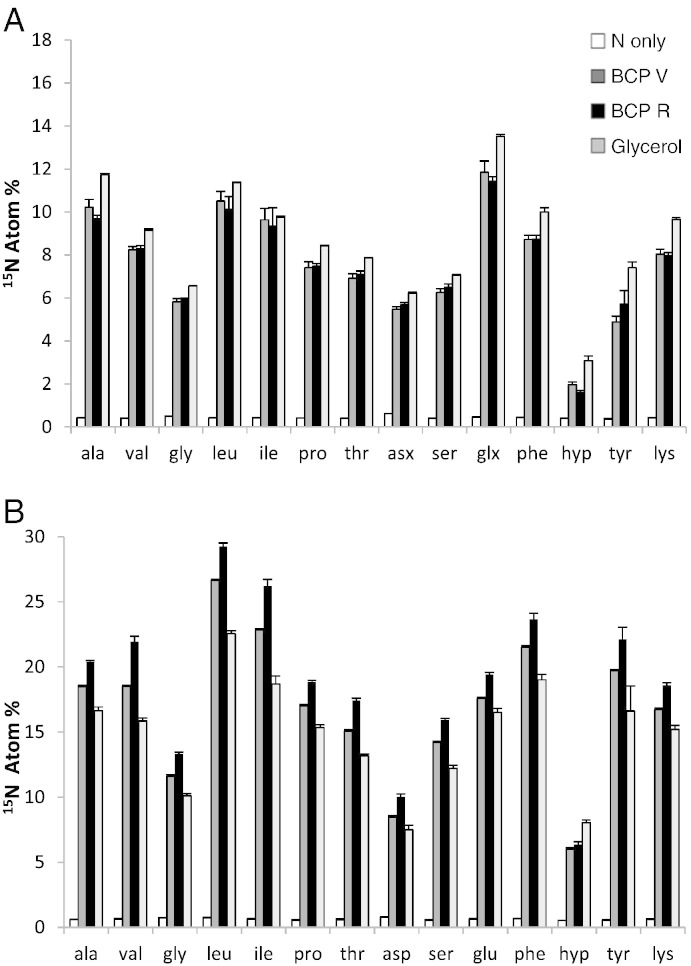
A: ^15^N incorporation into total hydrolysable amino acids (THAAs); l.s.d._α = 0.05_^15^N treatment effect = 0.23%. B: ^15^N incorporation into EPS amino acids (EPS-AAs); l.s.d._α = 0.05_^15^N treatment effect = 0.43%.

In the THAA pool ^15^N-labelling was greatest following the treatment with glycerol and declined in the order glycerol > BCP_V_ = BCP_R_ > N only (l.s.d. 0.23%; Fig. 2A). This was true for every AA residue. Mean enrichments (by treatment) were 8.70, 7.57, 7.54 and 0.44%, respectively. However, in the EPS, mean AA ^15^N enrichment was greatest for the BCP_R_ treatment and declined in the order BCP_R_ > BCP_V_ > glycerol > N only. This was true for every AA, except hydroxyproline, which was most enriched by glycerol addition (Fig. 2B). Mean enrichments were 18.82, 16.74, 14.82 and 0.63%, for BCP_R_, BCP_V_, glycerol, and N, respectively (l.s.d. 0.43%). The 3 AAs most enriched with ^15^N from addition of K^15^NO_3_ (all treatments) were hydrophobic residues: leucine and isoleucine (branched chain amino acids), and phenylalanine. Phenylalanine is a precursor of tyrosine which was also the 4th most enriched AA. The 3 least enriched were glycine, aspartate/asparagine and hydroxyproline. With regard to overall enrichment, the mean AA ^15^N enrichment was 6.06% in the soil total hydrolysable amino acid pool (THAA), and 12.75% in the EPS (with maxima of 13.51% and 29.24% ^15^N, respectively). ANOVA confirmed there was far greater proportional enrichment of the EPS vs. THAA (p < 0.001; Fig. 2A vs. B).

### 3.3 EPS colorimetric determinations and production efficiencies

**Fig. 3:**
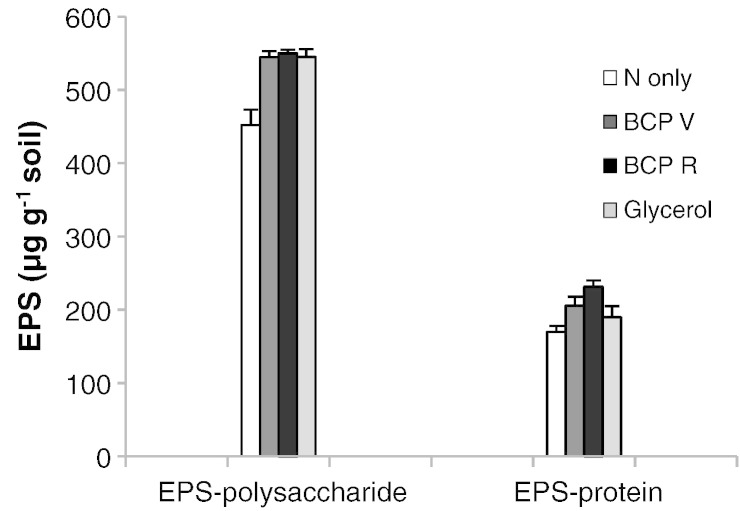
Total EPS-protein and EPS-polysaccharide determined colorimetrically. ANOVA treatment effect p = 0.025; l.s.d. EPS-protein = 36.70 μg g^− 1^ soil; l.s.d. EPS-polysaccharide = 41.44 μg g^− 1^ soil.

ANOVA of the quantitative colorimetric analysis of EPS-protein also showed statistically significant treatment effects (Fig. 3; p = 0.025). BCP_R_ caused the greatest increase of Lowry reactive EPS-protein of 61.9 μg g^− 1^ soil more than the ‘N only’ control (169.7 μg g^− 1^ soil). Considering the BCP treatments alone, only BCP_R_ resulted in a statistically significant increase in EPS-protein over ‘N only’ and glycerol (l.s.d._α = 0.05_ = 36.70 μg EPS-protein g^− 1^ soil). EPS-polysaccharide (as glucose equivalents) showed a more uniform response to C addition with greater EPS-polysaccharide measured with all treatments inclusive of C (ANOVA treatment effect p = 0.002). There were no statistically significant differences in extracellular polysaccharide produced between the two BCP's and glycerol (l.s.d._α = 0.05_ = 41.44 μg EPS-polysaccharide g^− 1^ soil).

The ATP concentration responses (Table 3) reflected the same pattern of observations as the THAA ^15^N % response shown in Section 3.2 (glycerol > BCP_V_ = BCP_R_ > N only) except that BCP_R_ caused a much smaller increase in ATP over the control (+ 1.8 nmol g^− 1^ soil) than the large ATP increase in soil amended with glycerol (+ 6.71 nmol g^− 1^ soil). Importantly this difference was statistically significant (Table 3; p < 0.05) demonstrating that glycerol favours growth of the microbial biomass (intracellular biomolecules). The resulting EPS-production efficiencies showed greatest EPS-protein and EPS-polysaccharide production per unit ATP with BCP_R_.

**Fig. 4:**
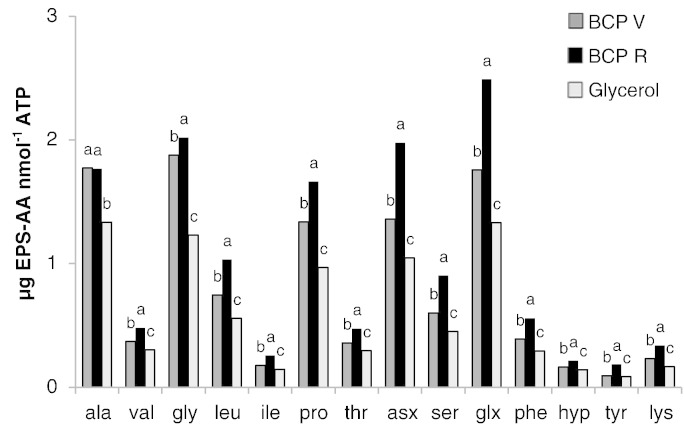
EPS-AA production efficiency. Data are provided on the natural scale. Treatment l.s.d. is calculated from transformed data: efficiency given the same letter is not significantly different (not for comparison between AAs). ANOVA of treatment effect p < 0.001.

As with colorimetrically determined EPS-protein efficiency described above, the substrate-induced EPS-AA efficiency was greatest when no labile C was provided. However, among the treatments delivering C – increasing both ATP and EPS –, BCP_R_ resulted in the highest EPS-AA production efficiency (mean = 0.96 μg EPS-AA nmol ATP^− 1^), followed by BCP_V_ and then glycerol (Fig. 4). The production efficiencies induced by organic amendment reassuringly match the response pattern for ^15^N enrichment (Fig. 2B), i.e., greatest for BCP_R_, followed by BCP_V_, then glycerol. For statistical comparison the data required transformation to stabilise residual variability (log_10_). Efficiency differences between treatments were all statistically significant at the 5% confidence level, except for alanine (Fig. 4). Supplementary Table 2 compares the transformed mean AA data on a numerical basis.

## 4 Discussion

### 4.1 Extraction and analytical methods

As with the more time-consuming analyses, the colorimetric protein assay showed that BCP_R_ caused statistically significant increases in EPS-protein (Fig. 3). Considering that methods of hydrolysis can be incomplete for hydrophobic peptide residues (Roberts and Jones, 2008) colorimetric analysis of non-hydrolysed extracts remains a justifiable approach and low cost option for measuring EPS-protein in soil. The colorimetric results for polysaccharide (Fig. 3) show that all substrate additions (inclusive of C) caused increase in extracellular sugars, and indicate that polysaccharides form the bulk of the EPS matrix. This corroborates observations made by Schlecht-Pietsch et al. (1994) who also observed an increase in total soil polysaccharide in response to substrate addition, but were unable to distinguish between extra- and intracellular polysaccharide at that time.

The amino acids measured in the total extractable pool (THAA) 10 days after treatment was applied accounted for more than half of the ^15^N provided. Considering only 14 AAs were quantified, and based on a peptide N content of about 15.5% (Schindler et al., 2007) provides reasonable evidence that most of the ^15^N provided (150 μg N g^− 1^ soil) had been biologically assimilated. This also shows that THAA extraction was highly efficient, which is in agreement with Roberts and Jones (2008) who stated that hydrolysis with HCl at around 100 °C extracts most intracellular biomolecules. Considering this high extraction efficiency, the THAA pool is also likely to represent most AAs constituting the extracellular microbial peptides. Moreover, comparison of ^15^N enrichment between hydrolysed EPS extracts (up to 29.2% ^15^N) with soil THAA (maximum 13.5% ^15^N) supports the premise that the THAA protocol extracts a larger proportion of pre-existing extracellular peptides with a natural abundance of ^15^N/^14^N (including pre-existing EPS and decaying organic matter).

Conversely, the EPS extraction (using CER) removed a greater proportion of recently synthesised biomolecules. Extraction with CER was proposed to be selective for the extracellular polymers of biofilms (Frolund et al., 1996). The absence of extraction-induced cell lysis has been supported by extracellular ATP analyses in extracts of sediments (Takahashi et al., 2009) and intracellular ATP measurements in soil (Redmile-Gordon et al., 2014b). The present work shows that the EPS extraction procedure selects for microbial products produced de novo (enriched in ^15^N) over the older pre-existing soil organic matter (SOM) which contained ^15^N at natural abundance. This selectivity for EPS produced de novo was initially proposed to be important by Redmile-Gordon et al. (2014b) who used a colorimetrically determined estimate of co-extracted humified SOM as an indicator of (un)suitability for other EPS extraction techniques. Naturally, such an approach cannot be said to be conclusive due to the lack of a clear chemical definition for humified SOM (and the limited number of soils investigated) but the ^15^N results discussed above provide much firmer support for this premise. The advantages of a technique which extracts newly synthesised EPS are that this facilitates scientific investigation by a) reducing the time required for experimental incubation, and b) minimising the risk of mis-allocating effects to EPS that may belong to some other co-extracted pool of SOM. For example, Gillespie et al. (2011) demonstrated that functional impacts were likely to be mis-allocated to fungal glycoproteins by the application of over-inclusive extractions with hot aqueous citrate. Finally, c) the measurement of EPS recently exuded is likely to be of highest functional relevance to the contemporary microbes producing it.

### 4.2 EPS is a more dynamic fraction of SOM

Using highly inclusive extraction methods (Jim et al., 2003; Knowles et al., 2010) the quantitative extracts of total hydrolysable soil amino acids (THAAs) did not show any treatment induced change in soil AA concentration (Fig. 1A). Total soil peptide can be remarkably stable, persisting for years due to physical or chemical stabilisation (Schmidt et al., 2011). However, EPS concentrations were highly responsive (Fig. 1B). The results presented here demonstrate that 1) peptides in microbial EPS are a more dynamic pool of soil organic matter than total soil peptide, 2) the EPS extraction targets this specific pool, and 3) this pool is linked to immobilisation of NO_3_-N. The measurable increase of AAs in the EPS found using GC/FID, and the demonstration that the EPS was serving as an external repository for immobilised nitrate (GC–C–IRMS data) offer intriguing prospects: 1) for subsequent microbial uptake, and 2) direct plant uptake. While AA-N has been predicted to comprise up to 90% of total N flux into maize (Jones and Darrah, 1994), Jones et al. (2013) subsequently reported that ^15^N from added glutamate was mostly taken up by plants after microbial mineralisation. Evidence concerning bioavailability is needed to test the assertion that EPS improves efficiencies of nutrient cycling (Flemming and Wingender, 2010) or conversely the possibility that this pool of organic N represents a route for C sequestration (e.g., Knicker, 2011; Cotrufo et al., 2013). In either case, longer term studies are required.

### 4.3 The BCP effect

Tracing the fate of ^15^NO_3_ improves our understanding of the mode of action underlying the biological retention of NO_3_-N in soil. The THAA ^15^N enrichment firstly showed that microbial assimilation of NO_3_-N had occurred with provision of all labile C substrates. However, the mechanisms of glycerol- and BCP-induced retention were demonstrably different: the hypothesis that BCP would foster greater proportional investment into extracellular polymers was validated, and comparison of the increases in EPS due to substrate addition shows that EPS-AA production efficiency was much greater when provided with BCP rather than glycerol (Table 3). BCP thus promotes EPS exudation (both proteinaceous EPS and exopolysaccharide) with BCP produced from reclaimed oils (BCP_R_) causing the greatest bias for exudation of AAs (Fig. 4). The BCP_R_ contained a higher content of fatty acid methyl esters and organic salts of potassium than either BCP_V_ or glycerol (Table 1) which may have contributed to the bias for EPS development and high production efficiencies per unit ATP (Fig. 4; Table 3). Furthermore, due to the higher C content, less BCP_R_ (total mass) was added than either BCP_V_ or glycerol (Table 1). Translation of these findings into agronomic practice may help to improve the overall efficiency of biodiesel production, with the greatest benefit arising when biodiesel is produced from reclaimed oils, with the BCP_R_ subsequently being used directly to augment reserves of soil EPS. While this extracellular matrix is likely to be important for maintaining soil structure for agronomic purpose (Or et al., 2007b) field studies are still clearly needed.

Addition of N alone caused only a very small increase in ^15^N of THAA, indicating C limitation of the microbial biomass. The propensity for glycerol to favour growth of microbial biomass might be expected from its common use as a substrate in batch cultures. However, Hilliou et al. (2009) found that glycerol was also suitable as a sole carbon source for the production of EPS-polysaccharides in batch reactors containing *Pseudomonas*, and Shemesh and Chai (2013) found glycerol was one of the most effective of a range of substrates for promoting biofilm formation in *Bacillus* spp. (albeit when coupled with high Mn availability). While glycerol may be a useful substrate for developing biofilms, the concentration of microbial ATP presented in Table 3 suggests that bias for growth of the microbial biomass occurred in this soil. Furthermore, in the THAA pool which includes the bulk of intracellular material, ^15^N was greatest in the AAs of soil given glycerol (Fig. 2A).

Among the AAs measured in the THAA fraction, Glx residues showed the greatest increase in ^15^N, which is in accordance with Geisseler et al. (2010) who stated that glutamine/glutamate (collectively Glx) are the main intracellular products of inorganic-N assimilation in soil. In contrast to the sum of glutamate and glutamine (Glx) in THAA, the complimentary N carriers: aspartate and asparagine (Asx) contained little of the added ^15^N (only Hyp residues showed less enrichment; Fig. 2A). Simultaneously, Asx was the most abundant AA residue in THAA (Fig. 1A). The low enrichment adds support to Payne's (1980) discussion that evolution has favoured the central role of Glx as a biological N carrier. Asx concentration in the EPS extracts was much lower (Fig. 1B) and featured even lower enrichment (Fig. 2B). The exact reasons for this are not clear and studies over time may be revealing.

### 4.4 Amino acids in the EPS

Leucine, phenylalanine, and tyrosine all show increased proportional abundance in EPS compared to THAA (Fig. 1A vs. B). Furthermore, all hydrophobic residues (Val, Leu, Ile, Phe, and Tyr) in EPS showed disproportionately high ^15^N enrichments (GC–C–IRMS; Fig. 2B) compared to the concentration increases (GC/FID). This could be either due to a) increased turnover, or b) the incomplete hydrolysis of hydrophobic residues leading to underestimation by GC/FID (Roberts and Jones, 2008). Stable isotope probing (SIP) can thus give more robust indications of the relative differences between quantitative AA syntheses in soil. Nonetheless, GC/FID results show that BCP_R_ caused the largest increases in concentrations of Leu, Phe, and Tyr in the EPS, with concentrations 70%, 61% and 153% higher than the control (N only), respectively. This more than doubling of EPS-tyrosine concentration through provision of BCP_R_ is intriguing because EPS has long been postulated to serve important roles in colony adhesion of biofilms (e.g., Flemming and Wingender, 2001) and tyrosine has repeatedly been implicated in adhesion and structural roles in the extracellular space of eukaryotes (e.g., Stone et al., 2009), and was recently proposed to play a mechanistic role in the aggregate stability of aerobic sludges (Zhu et al., 2012). The propensity for aromatic residues to aggregate through non-covalent π–π stacking is well reported (e.g., Chourasia et al., 2011). In soil, aromaticity (e.g., phenylalanine and tyrosine) was found to be prevalent in the hydrophobic fractions of soil extracts (Nebbioso and Piccolo, 2011) which also contained more stable macrostructures in solution (Nebbioso and Piccolo, 2011, 2012). These BCP-induced EPS-tyrosine increases therefore deserve closer attention in the context of improving soil physical characteristics.

BCP's potential to favour production of EPS over microbial growth is postulated to be of continued importance for the accumulation of highly water absorbent extracellular polymers in soil. This is because EPS exhibit both hydrophobic and hydrophilic properties (when soils are dry, and after a period of pre-exposure to water, respectively). Or et al. (2007b) describe this phenomenon and its potential significance for agriculture as it prevents osmotic stress and slaking of soil aggregates. Exopolysaccharide production was recently shown to be a process under positive feedback (Elsholz et al., 2014). In the aforementioned study, the EPS-polysaccharide exuded by *Bacillus subtilis* was found to also function as a signal to production — thus embodying a mechanism for positive feedback. This would be an evolutionarily favourable trait, as extracellular investment in EPS-polymers without a ‘switch’ to deactivate exudation when wasteful would be selected against (e.g., if extracellular biopolymers were being parasitised). Should this mechanism of positive feedback be ubiquitous in soils, maintenance of extracellular polysaccharide reserves may prove to be an important pre-requisite for the accumulation of organic matter in agricultural soils.

Interestingly, hydroxyproline (Hyp) was an exception: becoming most enriched in the EPS through treatment with glycerol. Hyp has been suggested to be involved in metal nutrient acquisition and competitive microbial allelopathy (Vranova et al., 2011). In the current experiment, microbial biomass was found to be greatest following glycerol treatment, and thus probably also was interspecific microbial competition. The increase in ^15^N enrichment was not coupled with an increase in the concentration of Hyp in EPS (from GC/FID). This could indicate increased turnover. Indeed, Hyp is hydrophilic which is a property said to contribute to higher bioavailability (Rothstein, 2010). Hyp was also found to be especially abundant in anti-listerial bacteriocins (Chakchouk-Mtibaa et al., 2014). If Hyp is considered an indicator of microbial allelopathy, then the present measurements lend support to the suggestion that EPS provides a direct competitive advantage to the organisms producing it (Flemming and Wingender, 2010). On a less conditional basis the present results concerning Hyp contradict the assertion of Philben and Benner (2013) that plants are the only major biochemical source of hydroxyproline in soil.

## 5 Conclusions

This work provides a clear demonstration using ^15^N stable isotope probing with GC/C/IRMS that microbial allocation of nitrate-N to EPS-AA is stimulated through provision of BCP, and most notably by BCP_R_. Both BCP's from biodiesel manufactured with recycled oils and virgin oils caused greater incorporation of the isotopically labelled NO_3_ into the EPS by a statistically significant margin compared to glycerol. The hypothesis that increased production of EPS-protein would occur in soil given BCP was also supported by simple colorimetric analysis of EPS-protein, with BCP_R_ again causing the greatest increase. The BCP_R_ treatment also resulted in the greatest EPS production efficiency per unit ATP (EPS-protein and EPS-polysaccharide). This measure was found to give greater sensitivity as an indicator of changing substrate quality as opposed to either total soil-EPS or THAA concentration. In all aspects, microbial EPS was shown to be more responsive to treatment than the more broadly defined and inclusive pool of SOM represented by THAA. The amino acids Ile, Leu, Phe and Tyr were more ^15^N-enriched in EPS than in bulk SOM. The fact that EPS production efficiency was greatest using BCP_R_ as a carbon source contributes to the already favourable LCA associated with the production of biodiesel from reclaimed oils over biodiesel crops grown only for fuel.

## Figures and Tables

**Table 1:** Composition of BCP's produced from i) waste cooking oil from a variety of restaurants (BCP_R_), and ii) virgin oilseed rape (BCP_V_).

Biodiesel Co-Product origin	Potassium hydroxide (% KOH)	Potassium soap (% oleate equivalent)	Fatty acid methyl esters (FAME; %)	Volatile organics (% at 105 °C)	Glycerol (%)	H_2_O (% before dilution)
BCP_R_	0.1	29.3	12.1	7.3	49.3	1.9
BCP_V_	2.3	22.3	0.2	10.8	62.6	1.8

**Table 2:** Treatment comparison.

Treatment name and carbon source	Substrate mass (mg)	C (%)	N as KNO_3_ (mg)	C/N	HCl (mg)	KCl (mg)	KOH (mg)	Water (mL)	Total mass applied per 33 g soil (dry weight)
BCP_R_	192	51.52	4.95	20	2.59	8.07	0.01	4.93	5.17
BCP_V_	253	39.17	4.95	20	6.53		0.01	4.93	5.23
Glycerol	253	39.13	4.95	20	–	13.36	–	4.93	5.23
‘N only’ (control)	–	–	4.95	–	–	13.36	–	4.93	4.98

**Table 3:** EPS production efficiency (from biomass-ATP and colorimetric analyses).

treatment	ATP (nmol g^− 1^ soil)	EPS-polysaccharide (μg EPS nmol^− 1^ ATP) l.s.d = 23.85	EPS-protein (μg EPS nmol^− 1^ ATP) l.s.d = 9.10
N only	4.49^c^	100.64⁎	37.81⁎
BCP_R_	6.29^b^	87.42^d^	36.82^g^
BCP_V_	8.47^ab^	64.31^de^	24.27^hi^
Glycerol	11.20^a^	48.65^e^	16.96^i^

^a,b,c,d,e,^^g,h,i^Means with the same letter are not statistically different (> l.s.d. of the logged data).

⁎ Not directly comparable (not provided with carbon).
