# Supplementary material for: Engineering soil organic matter quality: Biodiesel Co-Product (BCP) stimulates exudation of nitrogenous microbial biopolymers
Source: Geoderma. 2015 Dec;259-260:205–12. doi: 10.1016/j.geoderma.2015.06.006 (PMC4550076; doi:10.1016/j.geoderma.2015.06.006)
Supplement: Supplementary file 1 — Supplementary tables. [file mmc1.docx]

***Supplementary Table 1:*** *BCP_R_ Inorganic content (ppm). BCP_V_ not determined (values expected to be approximately equal to BCP_R_ since manufactured in the same facility).*

| Biodiesel Co-Product origin | As, Cd, Co, Cr, Se, Mo, Ni, Ti | Pb | Al |  | Ca | Cu | Fe | Na | P | S | Zn | Mn | Mg |
| --- | --- | --- | --- | --- | --- | --- | --- | --- | --- | --- | --- | --- | --- |
| BCP_R_ | <0.01 | 0.13 | 2 |  | 4 | 4 | 32 | 116 | 10 | 32 | 9 | <0.1 | <0.01 |
| STD_GRASS_ | <7.62 | 0.80 | 258 |  | 7613 | 6 | 501 | 1743 | 3126 | 1914 | 21 | 54.8 | 1417.80 |

***Supplementary Table 2:*** *Statistical comparison of EPS-AA production efficiencies (log_10_).*

| treatment | (µg EPS-AA nmol^-1^ ATP)  *l.s.d. = 0.031* |
| --- | --- |
| N only | -0.155* |
| BCP_R_ | -0.158 ^a^ |
| BCP_V_ | -0.289 ^b^ |
| Glycerol | -0.405 ^c^ |

All Data transformed log10

* not directly comparable (not provided with carbon)

a,b,c means with same letter are not statistically different (> l.s.d of the logged data)

***Supplementary Table 3:*** *Statistical comparison of EPS amino acid concentration in soil (log_10_ GC-FID data; µg g^-1^ soil). ANOVA for significance of treatment effects p = 0.024 (l.s.d. =* *0.095); ANOVA interaction (treatment x amino acid) p = 0.001.*

| Treatment | Mean log_10_ |
| --- | --- |
| BCP_R_ | 0.640 |
| BCP_V_ | 0.638 |
| Glycerol | 0.644 |
| N only | 0.497 |
